# When Childhood Trauma Meets War: Emotional Eating Through the Lens of PTSD

**DOI:** 10.5041/RMMJ.10550

**Published:** 2025-07-31

**Authors:** Zohar Spivak-Lavi, Yael Latzer, Orna Tzischinsky

**Affiliations:** 1 Department of Social Work, Yezreel Valley College, Emek Yezreel, Israel; 2 Faculty of Social Welfare and Health Sciences, University of Haifa, Haifa, Israel; 3 Eating Disorders Institute, Rambam Health Care Campus, Haifa, Israel; 4 Department of Behavioral Sciences, Yezreel Valley College, Emek Yezreel, Israel

**Keywords:** Childhood trauma, emotional eating, post-traumatic stress disorder (PTSD), trauma recovery, war exposure

## Abstract

**Objective:**

This study examines the interplay between childhood trauma, war exposure, and maladaptive coping mechanisms, with a focus on post-traumatic stress disorder (PTSD) as a mediator and war exposure as a potential moderator in the relationship between childhood trauma and emotional eating.

**Methods:**

Participants completed validated measures of childhood trauma, PTSD, and emotional eating. Statistical analyses included Pearson correlations, stepwise linear regression, and moderated mediation models, adjusting for age and gender.

**Results:**

The study included 426 Hebrew-speaking Israeli adults (52.8% female, 47.2% male, mean age 40). Childhood trauma, particularly emotional abuse, was significantly associated with PTSD and emotional eating. The relationship between childhood trauma and emotional eating was fully mediated by PTSD, with a stronger effect observed for emotional abuse. War exposure significantly predicted PTSD but did not moderate the link between PTSD and emotional eating. Sex differences emerged, with female participants exhibiting higher PTSD levels than males.

**Conclusions:**

The findings emphasize the enduring impact of childhood trauma, particularly emotional abuse, on maladaptive coping mechanisms like emotional eating, mediated by PTSD. While war exposure intensified PTSD symptoms, it did not significantly influence emotional eating. These results highlight the differential effects of early- and later-life traumas, offering insights for targeted interventions in trauma recovery.

## INTRODUCTION

Early experiences of childhood trauma have profound and enduring effects on mental health, greatly elevating the likelihood of developing psychological disorders later in life.^1^ These adverse experiences increase vulnerability to additional trauma throughout the lifespan, influencing one’s emotional and psychological state.^2^ The impact of trauma often manifests through various somatic and emotional symptoms such as emotional eating, difficulties in adaptability, and the development of post-traumatic conditions.^3^ In some cases, these symptoms can worsen in response to prolonged stress, such as during an ongoing war.^4^

### Childhood Trauma

Experiences of childhood trauma come in many forms and often leave deep emotional traces. Abuse and neglect are the most common categories referred to, but these terms cover a wide range of situations, some visible, others more hidden. These include physical violence, sexual harm, emotional degradation, and the absence of care, whether physical or emotional.^5^,^6^

Physical abuse can involve hitting, shaking, or burning, while sexual abuse refers to exposing a child to acts or situations that are sexually inappropriate.^1^,^7^ Emotional abuse, which can be harder to recognize, happens when a child is regularly criticized, rejected, or humiliated, causing lasting harm to their self-worth.^8^

A child who does not get enough food, a place to sleep, or medical help is considered physically neglected.^9^ Emotional neglect, on the other hand, relates to the child not having received enough love, attention, or emotional safety to promote healthy psychological development.^10^ These forms of trauma rarely appear in isolation. A child experiencing one type of abuse is likely to face others as well. The combination of different traumas can be much more damaging than each one alone.^11^,^12^ Studies have shown that having multiple traumatic experiences in childhood raises the chances of developing mental health problems later on.^1^

Emotional abuse is the type of trauma most often reported in the literature, with estimates pointing to around 36% of children being affected, of which physical abuse is reported in about 22% of childhood trauma cases, and sexual abuse in roughly 13%.^13^

Early trauma often disrupts the way children learn to manage emotions and stress.^14^ Without stable coping skills, some end up adopting patterns that are harmful over time.^15^ In adulthood, this might show up as self-injury, personality difficulties, or even psychotic symptoms in some cases.^16^

Additionally, trauma survivors frequently exhibit externalizing behaviors, such as conduct disorders, and internalizing conditions like depression, anxiety,^17^ and disturbed eating.^18^ Post-traumatic stress disorder (PTSD) is particularly prevalent among individuals who suffered emotional or sexual abuse or cumulative trauma during childhood,^19^,^20^ highlighting the profound and varied consequences of early-life trauma.^21^

### Post-traumatic Stress Disorder Following Childhood Trauma

When children are exposed to chronic or repeated forms of abuse and neglect, their chances of developing PTSD later in life increase significantly, particularly in cases involving sexual or emotional abuse. These early experiences, especially when complex and prolonged, can interfere with basic developmental tasks such as building emotional regulation and forming secure relationships.^19^

The prevalence of PTSD among adults reporting histories of childhood trauma ranges from 30% to 38%; rates are even higher among those exposed to multiple or overlapping forms of trauma.^22^ In clinical settings, the numbers are often much higher in individuals who underwent emotional or physical neglect—up to 60%.^23^ These figures highlight just how strongly early adversity can shape patterns of vulnerability in mental health later on.^22^ Post-traumatic stress disorder is often the mechanism through which exposure to trauma leads to broader psychological difficulties.^24^ Symptoms, such as intrusive memories, emotional numbness, heightened alertness, and avoidance behaviors, tend to disrupt emotional stability and can deepen the damage caused by the original trauma.^25^ This ongoing imbalance often sets the stage for additional challenges, including the development of maladaptive coping strategies.

A more common outcome in this context is emotional eating—a behavior that can emerge when individuals struggle to regulate distress.^26^,^27^ Emotional regulation difficulties are frequently rooted in early trauma, making it harder to manage daily stressors and increasing the likelihood of relying on food for comfort.^28^ In many cases, the connection between PTSD and eating behaviors reflects a broader pattern of emotional and psychological dysregulation that continues well into adulthood.

### Emotional Eating Stemming from Trauma

Emotional eating refers to a pattern whereby individuals seek food to cope with difficult emotions such as sadness, anxiety, stress, or anger, rather than in response to physical hunger.^29^ This behavior often involves overeating or making impulsive food choices, especially when under emotional strain.^30^ While it may offer momentary comfort, for those with a trauma history it can have serious long-term consequences, including weight gain and increased risk for obesity.^31^,^32^

Many emotional eaters are drawn to high-calorie “comfort foods,” especially when struggling to regulate their emotions.^33^ Post-traumatic stress symptoms linked to emotional dysregulation intensify this tendency, making trauma-exposed individuals particularly prone to using food as an effective coping mechanism.^34^ Although prevalence varies across populations, the impact of emotional eating can deepen when additional traumas occur later in life, further compounding psychological distress and maladaptive eating patterns.^35^

### Impact of Additional Trauma Later in Life

In many cases, individuals experience more than one traumatic event over the course of their lives, especially among survivors of childhood abuse, domestic violence, or mass violence.^36^ Exposure to sustained trauma or repeated exposure to multiple traumas is associated with a complex symptomatology that, in addition to the risk for developing PTSD symptoms, includes difficulties in emotional regulation, interpersonal functioning, and self-regulation.^36^

Childhood trauma—particularly events that occur in early life—plays a crucial role in long-term mental health outcomes.^37^ Individuals who have experienced multiple forms of childhood trauma are at increased risk for depressive symptoms and greater vulnerability to emotional distress in response to later-life traumas.^38^ This cumulative effect demonstrates how early-life trauma fundamentally alters the way individuals process subsequent stressors, amplifying their psychological impact and leading to more severe and persistent symptoms.^39^ While trauma experienced during adulthood places additional strain on coping mechanisms, childhood trauma tends to have a more significant influence, intensifying the psychological toll of later-life stressors.^40^

### The Impact of War and the Current Study

Exposure to war is recognized as one of the most significant stressors, with profound mental health effects on both civilian and military populations.^4^ Research highlights that war-related trauma increases the risk of developing psychopathologies such as PTSD and other mental disorders.^41^,^42^ Moreover, childhood trauma, including physical and emotional abuse, can exacerbate the effects of adult traumas, particularly prolonged war exposure, leading to long-term negative psychological responses in order to cope with emotional distress.^43^

Many studies have examined how cumulative childhood traumas affect the emotional functioning and well-being of adults.^44^ However, the current study emphasizes how cumulative traumas, starting with childhood trauma and continuing through war-related traumatic events in adulthood, shape emotional outcomes in adults. Since October 7, 2023, Israeli civilians have been enduring a prolonged traumatic situation, marked by continuous missile attacks, significant disruptions to daily life and routines, widespread loss of life, forced evacuations from affected areas, and the constant threat of further violence. Additionally, Israeli civilians, including women, children, and the elderly, have been held captive for an extended period. This has placed substantial psychological strain on the civilian population, exacerbating feelings of fear, uncertainty, and helplessness.

This study examined how the traumatic events of the October 7th war in Israel, characterized by extreme violence and fear, affected emotional functioning in adults, particularly among those with a history of childhood trauma. The focus was on how past and present traumas intersected, with emotional eating as a key outcome of interest.

We hypothesized that individuals who experienced trauma in childhood and were also exposed to the recent war would show greater emotional vulnerability. Specifically, we propose that PTSD symptoms mediate the relationship between childhood trauma and emotional eating in this population.

Based on this framework, the study hypotheses were:

1. Childhood trauma and exposure to war will be associated with higher levels of risk of PTSD symptoms, and emotional eating.
2. Childhood trauma (such as physical, sexual, or emotional abuse, and physical and emotional neglect) will predict the risk of developing PTSD symptoms, with exposure to war exacerbating the severity of PTSD symptoms among those who have experienced childhood trauma.
3. Risk for PTSD will mediate the link between childhood-trauma scores (total and subscales) and emotional eating: greater childhood trauma will be associated with higher PTSD severity, which in turn will predict higher emotional eating levels. This mediation is hypothesized to be moderated by war exposure, such that the PTSD-to-emotional-eating association becomes stronger as war exposure increases.

## METHODS

### Participants

Eligible respondents were Hebrew-speaking Israeli adults (≥18 years) registered with the iPanel online panel who provided informed consent and completed the baseline survey twice, on August 2023 (T1) and the follow-up in February 2024 (T2). Participants were excluded if they were under the age of 18 or not native Hebrew speakers. No participants were excluded for any other reason. All participants were informed that they could opt out of the study at any point.

The study participants were initially recruited in August 2023 through iPanel (https://www.ipanel..il), a major Israeli online platform with over 100,000 members. Panel members complete survey tasks in exchange for points, which can be redeemed for gift certificates. Between the two data collection time points, a war broke out in Israel on October 7, 2023, exposing the entire population to varying levels of direct and indirect war-related experiences. This temporal framework allowed for the examination of changes in psychological and behavioral outcomes in relation to war exposure. At T1, data on childhood trauma were collected using the Childhood Trauma Questionnaire (CTQ), while at T2, participants completed self-report questionnaires (detailed below) via the Qualtrics XM Platform (Qualtrics, LLC; Provo, UT, USA) for assessing PTSD and emotional eating. Sociodemographic details, including BMI, age, gender, marital status, education level, and employment status, were gathered as part of the same survey.

Prior to data collection, the study received ethical approval from the institutional review board (IRB, YVC2023-75; YVC-2024-38). All participants provided written informed consent, and their privacy and the confidentiality of the data were rigorously protected throughout the study. As the questionnaires involved recalling potentially distressing experiences, participants were informed that they could discontinue participation at any time without penalty. A list of professional mental health resources was also made available to all participants at the end of the survey. No reports of emotional distress were received. Accordingly, the data set includes complete data from all 426 participants, with no missing or partial data due to early withdrawal.

### Measures

#### Demographic data

Demographic details were collected through questions covering various background factors. These included age, marital status, employment status, and the degree of religious observance, which measured how closely individuals adhered to and practiced their religious beliefs.

#### The Childhood Trauma Questionnaire

The Childhood Trauma Questionnaire (CTQ) comprises 28 items covering the five types of childhood trauma: emotional abuse (EA), physical abuse (PA), sexual abuse (SA), emotional neglect (EN), and physical neglect (PN).^45^ Responses are rated from 1 (“never true”) to 5 (“very often true”). We calculated both total and subscale scores. According to standard guidelines, CTQ total scores of 25–50 indicate little or no trauma; 51–75, moderate trauma; 76–100, substantial trauma; and 101–125, severe trauma. A total score of 32 or higher is commonly used as the cut-off for identifying a clinically significant trauma history).^45^,^46^ Subscale scores were: 5–8, minimal; 9–12, mild/moderate; 13–15, substantial; and 16–25, severe.

#### The Post-Traumatic Stress Disorder Checklist

The Post-Traumatic Stress Disorder Checklist (PCL-5)^47^ for the *Diagnostic and Statistical Manual of Mental Disorders, Fifth Edition* (DSM-5) is a widely used self-assessment tool designed to evaluate PTSD symptoms in accordance with DSM-5 criteria. This questionnaire comprises 20 items scored on a 5-point Likert scale, from 0 (“not at all”) to 4 (“extremely”); higher total scores reflect more severe symptoms. The original version of the PCL-5 has shown exceptional reliability, evidenced by a Cronbach’s alpha of 0.94.^47^ A PCL-5 score of 33 or higher (out of 80) is generally recommended as the threshold indicating probable PTSD.

A Hebrew adaptation of the PCL-5 was validated in previous research^48^ and demonstrated strong reliability, with a Cronbach’s alpha of 0.92.

#### War Exposure

To evaluate the participants’ exposure to trauma, a self-report questionnaire was specifically developed for this study. The questionnaire addressed both direct trauma (e.g. experiencing life-threatening situations or witnessing acts of violence) and indirect trauma (e.g. hearing about a traumatic event affecting a close family member or friend) related to the events of October 7th.

These items were embedded within the demographic section and designed to capture both the objective nature and subjective emotional intensity of war-related experiences. While geographic proximity to conflict zones was not specifically assessed, the items reflected perceived personal exposure. The development of these items was guided by existing war exposure frameworks, though the instrument was not formally validated prior to use. The responses were rated on a Likert scale ranging from 0 (“not at all”) to 4 (“very much”), enabling quantification of the degree of exposure each participant experienced. The resulting scores provided a comprehensive measure of the participants’ trauma exposure levels.

#### The Dutch Eating Behavior Questionnaire

The Dutch Eating Behavior Questionnaire (DEBQ)^49^ is a widely used self-report instrument for evaluating three types of eating behaviors: emotional, external, and restrained eating. Emotional eating reflects eating triggered by emotions, while restrained eating involves consciously limiting food intake. External eating refers to eating in response to external cues, like the appearance or smell of food, rather than feelings of hunger. Comprising 33 items, it is scored on a Likert scale from 1 (“never”) to 5 (“very often”). The DEBQ has shown strong internal consistency, with Cronbach’s alpha values of 0.95 for emotional eating, 0.80 for external eating, and 0.93 for restrained eating. This questionnaire has been translated and validated in Hebrew, retaining similar reliability (Cronbach’s alphas of 0.94, 0.82, and 0.92 for the respective subscales).^50^

The Dutch Eating Behavior Questionnaire (DEBQ) does not have an official or clinically validated cutoff score.

Descriptive statistics for the study variables are reported in the Results section.

### Statistical Analysis

Continuous data were presented as mean and standard deviation, and categorical data as frequency and percentage. Pearson correlations were performed to assess the association between the study variables as well as the study variables with age. False discovery rate multiple comparison correction was used for multiple comparisons.

Stepwise linear regression was performed to predict PTSD using childhood trauma (total and the subscales) and war exposure as independent variables, adjusting for age and sex.

Moderated-mediation analysis (Model 14), adjusted for age and sex, was performed in SPSS v28 (IBM Corp., Armonk, NY, USA) with the PROCESS macro v3.1 (Andrew F. Hayes, https://processmacro.org). Childhood trauma total score (or its subscales) served as the independent variable, PTSD as a potential mediator of childhood trauma on emotional eating, and war exposure as a potential modifier of the mediator. Indirect effects were estimated with 5,000 bias-corrected bootstrap resamples to obtain 95% confidence intervals (CIs); an effect was considered significant when the CI excluded zero. Statistical significance was set at *P*<0.05.

## RESULTS

### Study Participants

A total of 426 people participated in the study. Full demographic data are provided in Table 1.

**Table 1:** Participant Sociodemographic Characteristics (*n*=426).

Characteristic	Value
Age (years), mean±SD (range)	40±10.5 (6–27)

Gender, *n* (%)	
Male	201 (47.2)

Female	225 (52.8)

Marital status, *n* (%)	
Married	262 (61.50)

In a relationship	46 (10.8)

Living with others	35 (8.2)

Single/living alone	51 (12.0)

Divorced/separated	32 (7.5)

Living arrangement, *n* (%)	
With partner/others	357 (83.8)

Alone	69 (16.2)

Education (years), mean±SD (range)	14.9±2.7 (6–27)

Employment, *n* (%)	
Employed	368 (86.4)

Unemployed	58 (13.6)

### Descriptive Statistics and Psychometric Properties

Table 2 summarizes means, standard deviations, ranges, clinical cut-off scores, the distribution of participants across CTQ-EA severity categories, and the proportion exceeding the PCL-5 cut-off. The DEBQ showed excellent reliability, with an overall Cronbach’s alpha of 0.95, and subscale values of 0.94 for emotional eating and 0.82 for external eating.

**Table 2:** Means, Standard Deviations, Ranges, and Cutoff Score for Study Questionnaires.

Parameter	Value	Range	Cutoff

Age, mean±SD, years	40±10.5	20–71	--

CTQ total, mean±SD	39.3±11.5	26–90	32

CTQ-pa, mean±SD	5.9±2.3	5–19	8

CTQ-ea, mean±SD	7.6±3.6	5–25	10

CTQ-sa, mean±SD	5.9±2.5	5–21	8

CTQ-pn, mean±SD	10.2±1.9	6–19	8

CTQ-en, mean±SD	9.7±4.4	5–23	15

PCL-5, mean±SD	17.6±16.8	0–78	--

War exposure, mean±SD	3.6±2.7	0–15	--

Emotional eating, mean±SD	2.6±0.9	1.1–4.9	--

PCL-5, *n* (%)	18 (6.8)	--	--

Emotional abuse		--	--
None to minimal (5–8), *n*	207	--	--
Low-moderate (9–12), *n*	38	--	--
Moderate-severe (13–15), *n*	10	--	--
Severe-extreme (16–25), *n*	12	--	--

All values are questionnaire scores (points) unless otherwise indicated. Age is reported in years. Emotional-eating values are means on a 1–5 Likert scale. PCL-5 values are means on a 0–4 Likert scale.

--, not applicable; CTQ, Childhood Trauma Questionnaire; CTQ-ea, CTQ Emotional Abuse; CTQ-en, CTQ Emotional Neglect; CTQ-pa, CTQ Physical Abuse; CTQ-pn, CTQ Physical Neglect; CTQ-sa, CTQ Sexual Abuse; PCL-5, Post-traumatic Stress Disorder Checklist for DSM-5; SD, standard deviation.

The CTQ demonstrated acceptable internal consistency (α=0.84 for the total score; subscales: EA, 0.85; PA, 0.85; SA, 0.89; PN, 0.62; EN, 0.40). The PCL-5 also exhibited excellent internal consistency, achieving a Cronbach’s alpha of 0.94.

### Zero-order Associations

Childhood trauma scores (total and subscales) and war exposure were positively correlated with PTSD; higher values on either measure predicted more severe PTSD. In addition, emotional abuse was positively correlated with emotional eating. The total childhood trauma score (CTQ total) was significantly correlated with emotional eating, which appeared to be driven primarily by the CTQ emotional abuse (CTQ-EA) subscale. War exposure, however, was not correlated with emotional eating. Total childhood trauma score and emotional abuse had a weak positive correlation with war exposure. Note that age was negatively correlated with PTSD and war exposure and not associated with emotional eating (see Table 3).

**Table 3:** Pearson Correlation Between the Study Variables and Age.

Variables		Age	CTQ Total	CTQ-pa	CTQ-ea	CTQ-sa	CTQ-pn	CTQ-en	PTSD	War Exposure	Emotional Eating
Age	*r*	1	0.037	0.066	−0.032	0.012	0.007	0.079	−0.132	−0.201	−0.064
	*P*		0.543	0.276	0.595	0.847	0.913	0.194	0.006	<0.001	0.191
		
CTQ total	*r*		1	0.790	0.851	0.614	0.725	0.828	0.341	0.067	0.145
		
	*P*			<0.001	<0.001	<0.001	<0.001	<0.001	<0.001	0.271	0.016
			
CTQ-pa	*r*			1	0.685	0.499	0.461	0.491	0.173	0.032	0.083
	*P*				<0.001	<0.001	<0.001	<0.001	0.004	0.599	0.170
				
CTQ-ea	*r*				1	0.442	0.451	0.591	0.405	0.078	0.194
				
	*P*					<0.001	<0.001	<0.001	<0.001	0.200	0.001
					
CTQ-sa	*r*					1	0.378	0.248	0.193	0.004	0.070
	*P*						<0.001	<0.001	0.001	0.943	0.249
						
CTQ-pn	*r*						1	0.627	0.240	0.077	0.074
						
	*P*							<0.001	<0.001	0.207	0.220
							
CTQ-en	*r*							1	0.252	0.057	0.103
	*P*								<0.001	0.344	0.089
								
PTSD	*r*								1	0.425	0.381
								
	*P*									<0.001	<0.001
									
War exposure	*r*									1	0.072
	*P*										0.139
										
Emotional eating	*r*										1
										
	*P*										

Only the upper triangle is shown; blank cells indicate redundant correlations.

CTQ, Childhood Trauma Questionnaire; CTQ-ea, CTQ Emotional Abuse; CTQ-en, CTQ Emotional Neglect; CTQ-pa, CTQ Physical Abuse; CTQ-pn, CTQ Physical Neglect; CTQ-sa, CTQ Sexual Abuse; *P*, raw *P-*value; PTSD, post-traumatic stress disorder; *r*, Pearson correlation coefficient.

### Predictors of PTSD

Following the analyses of associations among the primary study variables, we proceeded to conduct a moderated mediation analysis. This model, grounded in measurements from two distinct time points, is recognized in the literature as a rigorous method for evaluating predictive relationships.^51^ Childhood trauma, including the total score and all five subscales, as well as war exposure, were significant predictors of PTSD, after controlling for age and gender. Table 4 displays the PTSD prediction model with childhood trauma and war exposure as independent variables, with age and gender included as covariates. For each one-point increase in the total childhood trauma score, PTSD increased by 0.4 points (β, 0.44; standard error [SE], 0.080; 95% CI, 0.30–0.59). Similarly, for each one-point increase in war exposure, PTSD increased by 2.5 points (β, 2.47; SE, 0.32; 95% CI, 1.84–3.10). The total childhood trauma score accounted for 11.5% of the variance in PTSD, while war exposure accounted for 15.1% of the variance.

**Table 4:** PTSD Prediction Model Using Childhood Trauma and War Exposure as Independent Variables Adjusting for Age.

PTSD Prediction Model Using	Predictor	β (unstd)	SE	*P*	Partial R^2^
**CTQ Total**	Age	−0.101	0.083	0.23	0.014

	Gender	6.800	1.684	<0.001	0.072

	PTSD	0.445	0.073	<0.001	0.115

	War exposure	2.469	0.319	<0.001	0.151

	Model R^2^	—	—	—	0.575

	Model *F* (df1,df2)	—	—	<0.001	F(4,182)=61.68

**CTQ Physical Abuse**	Age	−0.093	0.088	0.29	—

	Gender	7.029	1.771	<0.001	—

	PTSD	1.095	0.386	0.005	0.029

	War exposure	2.573	0.335	<0.001	0.164

	Model R^2^	—	—	—	0.542

	Model *F* (df1,df2)	—	—	<0.001	F(4,182)=53.93

**CTQ Emotional Abuse**	Age	−0.067	0.088	0.46	0.011

	Gender	4.150	1.771	0.017	0.019

	PTSD	1.655	0.402	<0.001	0.056

	War exposure	2.430	0.335	<0.001	0.158

	Model R^2^	—	—	—	0.529

	Model *F* (df1,df2)	—	—	<0.001	F(4,182)=51.07

**CTQ Sexual Abuse**	Age	−0.072	0.089	0.42	0.010

	Gender	3.114	1.783	0.087	0.013

	PTSD	1.292	0.482	0.008	0.026

	War exposure	2.614	0.342	<0.001	0.161
	Model R^2^	—	—	—	0.514
	Model *F* (df1,df2)	—	—	<0.001	F(4,182)=48.14

**CTQ Physical Neglect**	Age	−0.097	0.086	0.25	0.013

	Gender	6.064	1.710	<0.001	0.057

	PTSD	0.724	0.361	0.046	0.020

	War exposure	2.375	0.324	<0.001	0.152

	Model R^2^	—	—	—	0.514

	Model *F* (df1,df2)	—	—	<0.001	F(4,182)=48.25

**CTQ Emotional Neglect**	Age	−0.054	0.089	0.55	0.008

	Gender	4.168	1.776	0.019	0.018

	PTSD	1.417	0.409	0.001	0.041

	War exposure	2.416	0.340	<0.001	0.156

	Model R^2^	—	—	—	0.516

	Model *F* (df1,df2)	—	—	<0.001	F(4,182)=48.65

β (unstd), unstandardized regression coefficient; Beta-not used but this is the standardized regression coefficient; *F*, *F*-statistic from the regression model (df1=model degrees of freedom, df2=residual degrees of freedom); *P*, model-adjusted significance level; Partial R^2^, proportion of variance explained by the predictor; PTSD, post-traumatic stress disorder; R^2^, coefficient of determination, proportion of variance explained by the independent variables; SE, standard error of the slope.

Similarly, in the model that used emotional abuse and war exposure as potential predictors, for every one-point increase in emotional abuse, PTSD increased by 1.7 points (β, 1.66; SE, 0.23; 95% CI, 1.20–2.11), and for every one-point increase in war exposure, PTSD increased by 2.4 points (β, 2.43; SE, 0.32; 95% CI, 1.81–3.05).

Emotional abuse explained 14.9% of the variance in PTSD, while war exposure explained 14.6% of the variance. Although age was not associated with PTSD, gender was a significant predictor: female participants had significantly higher PTSD levels compared to male participants.

### War-exposure Moderation and PTSD Mediation

Since the total childhood trauma score and emotional abuse were significant predictors of PTSD, we hypothesized that it could be a mediator of the relationship between trauma and emotional eating. However, after correcting for age and gender, war exposure did not significantly moderate PTSD severity (F(1,267), 0.32; *P*, 0.57).

Thus, we tested a model in which war exposure was entered as a main effect and PTSD served as a potential mediator of the link between childhood trauma and emotional eating. In this model, PTSD fully mediated the association between total childhood trauma and emotional eating (IE, 0.008; SE, 0.002; 95% CI, 0.004–0.014). Regarding subscales of childhood trauma, when emotional abuse was used in place of total trauma score, PTSD was found to fully mediate the relationship between emotional abuse and emotional eating (IE, 0.026, SE, 0.008; 95% CI, 0.012–0.044). This model explained 12.8% of the variance in emotional eating. However, when the rest of the childhood trauma subscales were used instead of the total trauma score, PTSD was not found to fully mediate the relationship between emotional abuse and emotional eating (see Table 5).

**Table 5:** Emotional-eating Prediction Models Using (A) CTQ Total and (B) CTQ-ea.

A. Predictor: CTQ Total	Direct Effects			Mediator			Moderated Mediator		

	β	SE	P	β	SE	P	β	SE	P
Age	−0.003	0.007	0.73	−0.001	0.007	0.87	−0.001	0.007	0.92

Gender	0.384	0.111	0.001	0.255	0.114	0.024	0.264	0.115	0.02

Total trauma score	0.011	0.005	0.021	0.004	0.005	0.48	0.004	0.006	0.52

War exposure	−0.013	0.023	0.58	−0.063	0.025	0.012	−0.066	0.024	0.007

PTSD	--	--	--	0.018	0.004	<0.001	0.017	0.005	<0.001

War exposure by PTSD	--	--	--	--	--	--	0.001	0.001	0.57

Model									
*F*		4.55			7.73			6.28	
*P*		0.001			<0.001			<0.001	
R^2^		0.063			0.126			0.178	

**B. Predictor: CTQ-ea**	**Direct Effects**			**Mediator**			**Moderated Mediator**		

	**β**	**SE**	***P***	**β**	**SE**	***P***	**β**	**SE**	***P***

Age	−0.001	0.007	0.85	−0.001	0.007	0.91	0.001	0.007	0.96

Gender	0.349	0.113	0.002	0.246	0.113	0.030	0.254	0.113	0.026

Emotional abuse	0.043	0.015	0.005	0.017	0.016	0.30	0.018	0.022	0.41

War exposure	−0.013	0.022	0.55	−0.061	0.025	0.014	−0.081	0.038	0.035

PTSD	--	--	--	0.018	0.004	<0.001	0.013	0.008	0.11

War exposure by PTSD	--	--	--	--	--	--	0.001	0.002	0.55

Model									
*F*		5.23			7.86			6.36	
*P*		<0.001			<0.001			<0.001	
R^2^		0.072			0.128			0.130	

--, not applicable; β, beta coefficient (the slope of the regression line); CTQ, Childhood Trauma Questionnaire; CTQ-ea, CTQ emotional abuse sub-scale; *F*, model *F-*statistic; *P*, model-adjusted significance level; PTSD, post-traumatic stress disorder; R^2^, coefficient of determination, proportion of variance explained by the independent variables; SE, standard error of the slope.

## DISCUSSION

The current study examined the psychological and behavioral consequences of childhood trauma and war exposure, specifically focusing on the interplay between these traumas in predicting risk factors for

PTSD and emotional eating. Previous research has consistently highlighted the enduring impact of early trauma on mental health, frequently leading to increased vulnerability to PTSD and maladaptive coping mechanisms. Data for this study were collected at two time points: prior to the onset of the recent October 7th war and approximately 6 months afterward, allowing for a temporal examination of these associations within a changing context of war exposure.

The main findings indicated that childhood trauma strongly predicted the risk for developing PTSD in adulthood, with the risk for PTSD serving as a significant pathway through which early trauma influences emotional eating behaviors. In addition, the same model was found in emotional abuse, which is a subtype of childhood trauma. War exposure was found to be positively associated with the risk for PTSD.

### Hypothesis 1

The first hypothesis posited that both childhood trauma and exposure to war would be linked to greater risk for PTSD and emotional eating. Higher overall childhood trauma scores were associated with increased PTSD symptoms in adulthood. Emotional abuse, in particular, was also linked to emotional eating. However, the results did not fully support this hypothesis. No significant association was found between the total childhood trauma score or the other subscales and emotional eating; only emotional abuse showed this pattern. War exposure was related to PTSD risk, but not to emotional eating.

These findings aligned with existing literature and suggest that childhood trauma increases vulnerability to develop PTSD later in life.^52^ The specific association between emotional abuse and emotional eating supports the theory that trauma-related responses are often triggered by stimuli that resonate with the nature of the original trauma.^53^ While cumulative trauma literature emphasizes that multiple traumas, even if of different types, can amplify the traumatic experience,^54^ the distinct link between emotional abuse and emotional eating may stem from the subjective and complex nature of emotional abuse as a broad and somewhat ambiguous construct.^55^ This subjectivity could contribute to its unique association with emotional eating as a coping mechanism. In such cases, emotional eating may serve as a means not only to address physical hunger but also to mitigate emotional distress and reduce physiological stress responses.

Moreover, emotional eating in response to emotional abuse may reflect a specific mechanism for managing childhood trauma,^53^ indicating that emotional abuse may impact later emotional regulation abilities, which in turn may influence emotional eating as part of a more complex coping process. On the other hand, although war exposure was associated with PTSD, consistent with research literature,^56^ it did not correlate with emotional eating. This finding suggests that responses to traumatic events are not uniform; while childhood trauma, or at least certain aspects of it, was associated with emotional eating, war trauma—a different trauma type—was not. This points to a diverse and trauma-specific pattern of response, cautioning against generalizing trauma exposure and response patterns across different trauma types.

Although war exposure is a significant stressor, it may not activate the same emotional regulation mechanisms disrupted by early interpersonal trauma. Emotional eating may reflect long-standing coping patterns shaped by early, relational adversity—patterns that remain relatively stable and less reactive to later, non-relational stressors. This could explain why the PTSD–emotional eating link was not moderated by war exposure in this study.

### Hypothesis 2

Our second hypothesis focused on the connection between childhood trauma and PTSD symptoms, with the thought that war exposure would make PTSD even more severe for those with trauma histories. The results supported this: both childhood trauma and war exposure were significantly related to PTSD levels. People who experienced any type of childhood trauma reported more intense PTSD symptoms.

Our findings also pointed to the cumulative effect of early and later trauma. Participants who had undergone both childhood trauma and recent war exposure showed greater psychological distress,^57^ supporting other research that early trauma can make people more reactive to later stressful events, including war.^58^ Emotional abuse stood out, but in our study all trauma types were related to PTSD vulnerability.

One explanation is that early trauma leaves people with a lingering sense of helplessness or fear, which can resurface during a crisis. In the current war in Israel, civilians have been exposed to events that shake basic assumptions about safety, events such as displacement, violence, threat to life, and the loss of family members. Additionally, political and social tensions compound the situation. Since these stressors are ongoing and without clear resolution, they tend to intensify the emotional burden.

### Hypothesis 3

Our third hypothesis posited that PTSD would mediate the relationship between childhood trauma, including both the total score and subscales, and emotional eating. It further posited that war exposure would moderate this mediation, with the expectation that individuals with higher levels of exposure would show a stronger link between PTSD symptoms and emotional eating. Post-traumatic stress disorder was found to fully mediate the relationship between total childhood trauma and emotional eating, as well as the relationship between emotional abuse and emotional eating. However, contrary to our hypothesis, war exposure did not significantly moderate the link between PTSD and emotional eating. This finding remained unchanged even when including age and gender as covariates.

The relationship between childhood trauma and emotional eating, with PTSD serving as a mediator, was evident both for the overall CTQ score and specifically for the emotional abuse subscale. The relationship noted between emotional eating and emotional abuse further supports and strengthens the existing research literature.^59^ This may indicate that individuals with a history of childhood trauma, particularly those who have experienced emotional abuse, may adopt patterns of emotional eating as a coping mechanism^60^ facilitated by PTSD symptoms.^61^ It is important to emphasize that emotional abuse is often insidious and difficult to identify, both by others and by the individuals experiencing it. Unlike physical or sexual abuse, it frequently lacks visible signs or a clear narrative, making emotional abuse harder to name, process, or even recognize.^62^,^63^ Emotional eating may emerge as a silent expression of this form of trauma, a way of giving voice to pain that was never acknowledged and that may have remained unspoken. In this sense, eating becomes not only a means of emotional regulation, but a symbolic act that embodies distress that has no language. This could help explain why emotional abuse, more than other forms of childhood maltreatment, was uniquely linked to emotional eating in our findings.

While these patterns were statistically supported through the mediation model, it is important to note that the effect sizes, particularly for the total childhood trauma score, were small. This may reflect the complexity of emotional eating as a coping mechanism and its multifactorial nature. Nonetheless, even modest effects may hold clinical relevance when considering trauma-informed interventions at the population level.

### Central Mediating Role of PTSD

Our findings emphasized the critical role of PTSD in linking early traumatic experiences to later maladaptive coping behaviors,^64^ with emotional abuse standing out as a unique trauma subtype that can heighten susceptibility to emotional eating through elevated PTSD symptoms.^55^

In contrast, and contrary to our hypothesis, war exposure did not significantly moderate the relationship between PTSD and emotional eating, even when controlling for age and sex. The absence of a moderating effect from war exposure may imply that the connection between risk for PTSD and emotional eating is more strongly influenced by early childhood trauma than by subsequent trauma exposure in adulthood, such as war.^65^ This highlights the enduring and substantial impact of formative, early-life trauma on coping behaviors,^37^ suggesting that emotional eating as a response to stress is deeply rooted in past childhood experiences,^60^ while the role of more recent traumatic events may be comparatively less pronounced.

An additional explanation may be related to the possibility that a history of trauma can be reactivated and intensified in stressful situations that more closely resemble past childhood trauma rather than events like war.^66^ Such events may serve as activators of previous trauma. While the literature suggests that an accumulation of stress-inducing events can heighten or increase the risk of PTSD,^67^,^68^ it seems that war exposure did not act as a risk factor for childhood trauma survivors—and may even have contributed to their resilience. This potential resilience may stem from the capacity of individuals with a history of severe early-life trauma to develop a degree of psychological fortitude when faced with subsequent, non-similar stressful events later in life, since they may not evoke the same intense, trauma-specific responses associated with the initial childhood trauma.^66^

## PRACTICAL AND CLINICAL IMPLICATIONS

The findings of this study offer a number of meaningful implications for both clinical work and public health. First, the central role of PTSD as a mediator between childhood trauma, especially emotional abuse and emotional eating, underscores the importance of early identification and treatment of trauma symptoms. When PTSD is addressed among individuals with a history of emotional abuse, there may be a better chance of preventing the development of maladaptive coping strategies such as emotional eating.

Second, the results suggest that treatment approaches should be adapted to the nature and history of trauma. Emotional eating may reflect an entrenched pattern of coping that has its roots in emotional experiences that were either invalidated or never fully recognized. Therapeutic work that incorporates skills for emotional regulation, alongside space to reconstruct the personal narrative, may be especially helpful for these individuals.

Third, no sex differences were found in emotional eating in this study; women did report significantly higher PTSD symptoms than did men. This highlights the need for sex-sensitive approaches in clinical assessment and intervention. Sociocultural pressures, particularly those related to emotional expression and body image, may place women at greater vulnerability, and these factors should be considered in both diagnosis and treatment planning. Such pressures may also contribute to the way emotional eating develops and manifests in women, even if not statistically evident in this particular sample.

Fourth, in the public health sphere, our findings point to the importance of distinguishing between individuals who carry early trauma, especially emotional trauma, and those who are reacting primarily to a collective traumatic event such as war. Even when symptoms may appear similar, the underlying emotional sources are often very different and require different kinds of support. This distinction is particularly relevant now, in light of the current crisis in Israel. The Israeli context is characterized by strong social cohesion and a culture that places high value on family ties, interpersonal relationships, and mutual support. These elements often serve as informal support systems, particularly in times of collective distress, and may play a buffering role in how trauma is processed.

Still, these insights are not unique to the Israeli context. They can be applied to other populations facing collective trauma, whether it stems from war, natural disasters, or other large-scale emergencies. Recognizing the nature of the trauma can help guide more accurate and effective responses, both in clinical settings and in community-level mental health planning.

## LIMITATIONS AND FUTURE DIRECTIONS

The study acknowledges several limitations that should be considered when interpreting the findings. First, the sample was composed exclusively of Hebrew-speaking Israeli participants, which may limit the generalizability of the results to broader populations and cultural contexts. However, it is worth noting that Israel is a multicultural society where approximately 80% of citizens speak Hebrew, which may somewhat mitigate this limitation. Additionally, data collection relied on self-report measures, which may introduce biases such as recall bias and social desirability effects. The cross-sectional nature of the data collection limited insights into long-term changes or causations in trauma-related outcomes.

A second limitation concerns the way war exposure was measured. The questions related to exposure to the October 7th events were developed specifically for this study to assess whether and to what extent each participant was affected, either directly or indirectly. However, it is possible that our approach did not fully capture the complexity of multiple, simultaneous war exposures, both on the home front and in combat zones. Future studies should aim to develop and validate instruments that more accurately assess diverse and overlapping exposures to war-related events.

Future research should focus on exploring resilience factors that may mitigate the impact of childhood trauma and promote adaptive outcomes. Understanding what enables some individuals to adapt and grow despite early trauma can provide valuable insights for targeted interventions. Beyond emotional eating, investigating alternative and positive coping mechanisms such as social support networks, mindfulness practices, and other adaptive behaviors can broaden our knowledge of recovery pathways. Such insights could inform therapeutic approaches aimed at enhancing resilience and fostering long-term well-being in trauma survivors. A significant strength of this study is that the extreme collective experience faced by the Israeli population provides valuable insights into acute collective crisis situations. In addition, future studies could examine whether other maladaptive coping behaviors, such as substance use or dissociation, are similarly mediated by PTSD.

## CONCLUSION

This study underscored the profound impact of childhood trauma on later psychological outcomes, particularly in increasing the risk for PTSD and its role as a mediator for maladaptive behaviors such as emotional eating. The specific link between emotional abuse and emotional eating, mediated by PTSD, highlights the unique and enduring influence of early-life emotional trauma. While war exposure was positively associated with PTSD, it did not significantly influence emotional eating, suggesting that early-life traumas exert a more substantial influence on such coping behaviors than do later-life traumatic exposures like war. Importantly, the findings also indicated that childhood trauma, despite its potential for negative outcomes, may contribute to the development of resilience and post-traumatic growth when a person is faced with later-life traumas.

This perspective emphasizes the dual role of early trauma as both a risk factor and a potential catalyst for positive adaptation and coping, providing valuable insights for therapeutic approaches aimed at fostering growth alongside trauma recovery.

## Abbreviations

CI(s): confidence interval(s)
CTQ: Childhood Trauma Questionnaire
DEBQ: Dutch Eating Behavior Questionnaire
DSM-5: Diagnostic and Statistical Manual of Mental Disorders, Fifth Edition
IE: indirect effect
PCL-5: PTSD Checklist for DSM-5
PTSD: post-traumatic stress disorder
SPSS: Statistical Package for the Social Sciences.
